# Transcription Factor and lncRNA Regulatory Networks Identify Key Elements in Lung Adenocarcinoma

**DOI:** 10.3390/genes9010012

**Published:** 2018-01-05

**Authors:** Dan Li, William Yang, Jialing Zhang, Jack Y. Yang, Renchu Guan, Mary Qu Yang

**Affiliations:** 1 Joint Bioinformatics Graduate Program, Department of Information Science, George W. Donaghey College of Engineering and Information Technology, University of Arkansas at Little Rock and University of Arkansas for Medical Sciences, 2801 S. University Ave, Little Rock, AR 72204, USA; dxli@ualr.edu (D.L.); wxyang1@ualr.edu (J.Y.Y.); rxguan@ualr.edu (R.G.); 2 School of Computer Science, Carnegie Mellon University, 5000 Forbes Ave, Pittsburgh, PA 15213, USA; wyang1@andrew.cmu.edu; 3 Department of Genetics, Yale University, New Haven, CT 06520, USA; jialing.zhang@yale.edu

**Keywords:** transcription factor, long non-coding RNA, gene regulation, cancer, network, systems biology

## Abstract

Lung cancer is the second most commonly diagnosed carcinoma and is the leading cause of cancer death. Although significant progress has been made towards its understanding and treatment, unraveling the complexities of lung cancer is still hampered by a lack of comprehensive knowledge on the mechanisms underlying the disease. High-throughput and multidimensional genomic data have shed new light on cancer biology. In this study, we developed a network-based approach integrating somatic mutations, the transcriptome, DNA methylation, and protein-DNA interactions to reveal the key regulators in lung adenocarcinoma (LUAD). By combining Bayesian network analysis with tissue-specific transcription factor (TF) and targeted gene interactions, we inferred 15 disease-related core regulatory networks in co-expression gene modules associated with LUAD. Through target gene set enrichment analysis, we identified a set of key TFs, including known cancer genes that potentially regulate the disease networks. These TFs were significantly enriched in multiple cancer-related pathways. Specifically, our results suggest that hepatitis viruses may contribute to lung carcinogenesis, highlighting the need for further investigations into the roles that viruses play in treating lung cancer. Additionally, 13 putative regulatory long non-coding RNAs (lncRNAs), including three that are known to be associated with lung cancer, and nine novel lncRNAs were revealed by our study. These lncRNAs and their target genes exhibited high interaction potentials and demonstrated significant expression correlations between normal lung and LUAD tissues. We further extended our study to include 16 solid-tissue tumor types and determined that the majority of these lncRNAs have putative regulatory roles in multiple cancers, with a few showing lung-cancer specific regulations. Our study provides a comprehensive investigation of transcription factor and lncRNA regulation in the context of LUAD regulatory networks and yields new insights into the regulatory mechanisms underlying LUAD. The novel key regulatory elements discovered by our research offer new targets for rational drug design and accompanying therapeutic strategies.

## 1. Introduction

Lung cancer is one of the leading causes of morbidity and mortality worldwide, especially in men and smokers. In fact, according to an American Cancer Society report in 2017, one in four cancer deaths are from lung cancer [1]. It has been estimated that there will be approximately 222,500 new lung cancer cases and 155,870 lung cancer-related deaths in the United States in 2017. Lung adenocarcinoma is a major subtype of non-small cell lung cancer that accounts for 85% of lung cancer cases.

The initiation and progression of lung cancer is a complex process due to a number of factors, including environmental exposure [2], smoking [3,4], signaling pathways, as well as genomic variance. Previous studies have even discovered some genomic elements and suggest the involvement of genetic mutations [5,6], transcriptional dysregulation [7] , and immunosuppression [8]. Additionally, long non-coding RNAs (lncRNAs) have also been discovered to play critical roles in the development of various types of cancers [9,10,11,12,13].

Transcription factors (TFs) play critical roles in regulating the expression of genes [14]. Recent studies have shown cancer stem cells (CSCs) to be a potential cause of lung cancer initiation and development [15,16], while TFs to be important markers of CSCs [15]. The study of TFs has also improved our understanding of the mechanisms involved in the dysregulation of gene expression in diseases. For example, researchers investigated the transcription factor NF-κB, a driver of small cell lung cancer progression in mice, by assessing its expression and regulation patterns. The results indicated its overexpression in metastatic high-grade neuroendocrine lung tumors [17].

Additionally, several computational studies have used interactions and gene expression to identify disease-associated TFs and their functions in lung cancer. For example, the interactions between TFs and microRNAs were useful for discovering the regulatory roles of TFs in lung cancer [18].The expression patterns of the microRNAs, TFs, and the common genes regulated by them were used to construct lung cancer regulatory networks [19]. Currently, network-based methods are performed for studying cancer, including lung cancer. Research using co-expression analysis [11,20] identified gene modules with functional variations between different cancer conditions. Another approach combining multiple data sources to identify key genetic elements in breast cancer was also reported [21]. However, the regulatory patterns including the interactions between lncRNAs and TFs in lung cancer remain to be elucidated. More comprehensive studies are needed to address the divergent molecular mechanisms underlying lung cancer.

In this study, we focused on the identification of key regulatory elements, including TFs and lncRNAs, responsible for lung adenocarcinoma (LUAD) progression. An integrative systems biology approach combining somatic mutations, gene expression profiles, DNA-methylation, protein–lncRNA interactions, and TF–gene interactions was developed for identifying the regulatory networks in LUAD. Genes with similar expression patterns were clustered as gene modules by co-expression network analysis. We further used a Bayesian network approach to indicate the potential regulatory relationships (directed edges) between genes. The final regulatory networks were achieved by incorporating known TF and target interactions based on experimental evidence and curation. TFs and lncRNAs with driver somatic mutations and/or connected with regulatory networks enriched with TF target genes were further investigated as key regulatory candidates. Aside from the lung cancer-related key regulators that we identified and thoroughly investigated in our study, we also discovered several novel TFs and lncRNAs that we found to be key regulatory elements. Pan-cancer analysis of the transcriptomes from 16 solid-tissue cancers provided additional evidence of the functional roles of these key lncRNAs. In this study, we introduced a new comprehensive network-based analysis merging multi-layer genomic data for revealing regulatory relationships between genes in LUAD. The gene regulatory networks and key regulatory TFs and lncRNAs are now available for studies investigating lung cancer.

## 2. Materials and Methods

### 2.1. Multidimensional Genomics Datasets

We obtained RNA sequencing (RNA-seq) data, DNA methylation data, and somatic mutation profiles of LUAD from The Cancer Genome Atlas (TCGA, March 2017) data portal [22]. The data comprehensively represented 464 LUAD patients. As some patients had more than one sequenced sample, the number of LUAD tissue samples was actually larger than the total number of patients. We used all the samples available for each data type. Overall, we collected 540 RNA-seq, 527 DNA methylation, and 540 somatic mutation datasets for our analysis.

The RNA-seq data were generated from 56 normal and 484 LUAD tissue samples. Both raw reads and normalized gene expression (Fragments Per Kilobase of transcript per Million) data were downloaded from the TCGA data portal for our study. We used the raw reads for the differential expression analysis (DEG) and the normalized data for co-expression module discovery. The somatic mutations were called by Mutect2 [23]. The Methylumin Bioconductor package [24] was applied for normalization and calculation of the β values for DNA methylation data processing. The β values represent the ratio of the methylated probe intensity to the overall intensity at each CpG locus [25]. We filtered out the methylation samples that had no β values or no CpG island information or methylations that occurred beyond 1 kb upstream or downstream of the gene transcription start site (TSS). As a result, 446 DNA methylation samples consisting of 26 normal and 420 tumor samples were retained for the subsequent analysis.

### 2.2. Differential Analysis

We applied the edgeR Bioconductor package [26] to perform the differential expression analysis based on the RNA-seq data. The fold change (FC) and false discovery rate (FDR) were employed as the criteria for selecting the differentially expressed genes. After excluding the transcripts with median expression levels less than 0.5 FPKM (Fragments Per Kilobase of transcript per Million), the transcripts with |log_2_(FC)| > 0.5 and FDR < 0.05 were chosen as differentially expressed genes. For DNA methylation, we used samr R package [27] to perform the differential methylation analysis.

### 2.3. Regulatory Network Building

**Figure 1:**
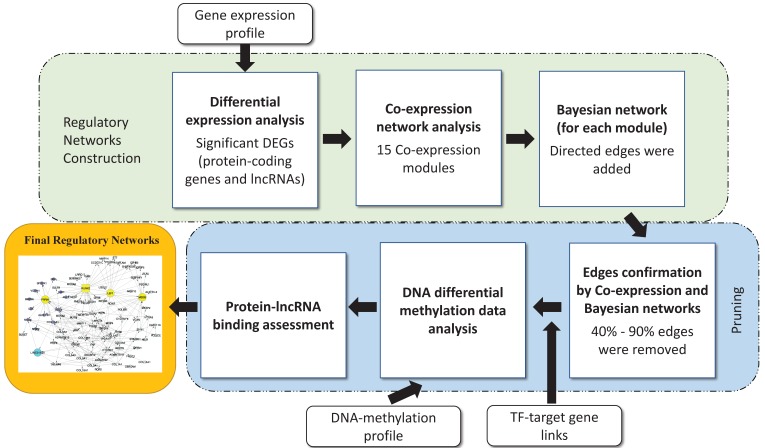
Gene regulatory network identification procedure. The procedure includes co-expression identification, edge determination by transcription factor (TF) targets, Bayesian analysis, and long non-coding RNAs (lncRNA)–protein binding potential. The DNA methylation analysis was used to infer potential epigenetic regulations. DEGs: Differential Expressed Genes.

The regulatory networks were obtained from multi-step analysis including co-expression module identification, Bayesian network analysis, TF target assessment, and further pruning to reduce false positives. The gene regulatory network identification procedure is illustrated in Figure 1.

First, we identified co-expression modules using weighted correlation network analysis (WGCNA) [28] based on RNA-seq expression profiles. The WGCNA algorithm yielded a Topological Overlap Measurement (TOM) and provided a generalized assessment of the edges between two gene nodes.

Then, Bayesian network analysis was conducted to infer potential regulatory relationships among genes in the co-expression modules. A Bayesian network is an NP-hard (non-deterministic polynomial-time hardness) problem [29] and often applies heuristic searching methods to reduce the search space size and computational complexity. Here, we used the hill climbing search approach to infer the direction of gene edges in the modules. This score-based optimization learning algorithm ranked the network structures according to a goodness-of-fit score and then identified the best network structures [30]. We used the bnlearn R package [31] to perform the analysis and measured the strength of the directed edges in the network. Then, all the edges were sorted in descending order of strength, and only those whose edges equaled 75% and above were retained. Additionally, edges whose connections were not confirmed by the co-expression were removed.

Additional regulatory relationships among genes were inferred based on TF and target gene interactions. We downloaded the TFs from four major databases, including JASPAR [32], AnimalTFDB 2.0 [33], Regulatorycircuits [34], and the Transcriptional Regulatory Element Database (TRED) [35]. The downstream target genes were selected based on a recent gene regulation study, and only lung cancer-specific TF and target interactions were used for network construction [34]. If a transcription factor and its target gene presented in the same module, a directed edge was added between the two gene nodes.

Each gene module contained protein-coding genes and lncRNAs. We calculated the binding scores to assess the potential binding between the lncRNAs and proteins [36]. The higher the score, the greater the potential that an lncRNA binds to a protein. Only interactions with binding scores equal to 25 and above (considered as real binding) were retained.

DNA methylation, perhaps because it blocks the promoters where transcription factors bind, was believed to play a crucial role in repressing gene expression. It has been observed that higher DNA-methylation in promoters corresponds to the lower expression of the corresponding genes. Therefore, we removed the edges between TF and target genes if target gene expression was correlated with its DNA methylation in the promoter.

We applied a bootstrapping method to assess the robustness of the networks to noise. In each bootstrapping iteration, we randomly deleted 2% of the nodes from the networks. We then evaluated the percentage of preserved edges (regulation relationships) after 100 iterations for each network.

### 2.4. Driver Somatic Mutation Identification

The somatic mutation profiles from LUAD patients were obtained from the TCGA data portal. All the identified somatic mutations were merged into a single VCF file. We then used the Cancer-Related Analysis of VAriants Toolkit (CRAVAT 4.3) [37] to identify genes that harbored significant somatic mutations. CRAVAT combines two driver mutation predictors, CHASM [38] and VEST [39], to score the somatic mutations. Both the CHASM and VEST predictions were based on the Random Forest model and yielded *p*-values that were used to rank the significance of the somatic mutations in LUAD.

### 2.5. Gene Enrichment Analysis

The odds ratios of the target genes of a specific TF inside the network and outside the network were calculated. Next, we applied the Fisher Exact Test to assess the statistical significance of the target gene enrichment in the individual regulatory networks. We applied DAVID [40] analysis to evaluate the pathway enrichment of the genes in the regulatory networks. The networks were plotted using Cytoscape v3.4.0 [41].

## 3. Results

### 3.1. Differential Analysis of Multidimensional Genomic Profiles of Lung Adenocarcinoma

RNA-seq data generated from 56 normal and 484 lung adenocarcinoma tissue samples were obtained from the TCGA project. Differential expression analysis yielded 6220 differentially expressed genes, including 5934 protein-coding genes and 286 lncRNAs, (|log_2_(FC)| > 0.5 and FDR < 0.05), where 78 lncRNAs and 2165 protein-coding genes were under-expressed in tumors. We also obtained the DNA methylation profile generated from 26 normal and 420 tumor samples from the same patient cohort. Differential methylation analysis revealed 1903 and 2992 genes had positive (hyper-) and negative (hypo-) methylation, respectively, in their promoter regions (*q*-value ≤ 0.0075 and FDR < 0.05). We found two under-expressed lncRNAs and 281 protein-coding genes that had elevated methylation levels in their promoters. Furthermore, based on the somatic mutation profiles of the same LUAD patients, we found that 2835 genes harbored at least one significant somatic mutation (FDR < 0.05).

### 3.2. Disease Regulatory Network Identification

We conducted a co-expression analysis of with the differentially expressed genes in LUAD and revealed 15 co-expression gene modules. The modules contained 4012 differentially expressed protein-coding genes and 124 lncRNAs. Genes with similar or opposite expression patterns were clustered into the same module. TFs control target gene expression by interacting with *cis*-regulatory regions around these genes. To identify inference due to regulatory relationships among genes in the modules, we first searched for TFs and found that 297 of 1806 TFs from four major TF databases were present in the modules. The lung cancer-specific target genes of each TF were based on the annotation from a recent large-scale study [30]. Whenever a TF and corresponding target gene were present in the same module, we added a directed edge between them to account for the physical interaction and expression correlation. Additional edges were added by coupling Bayesian network and co-expression analyses. It has been widely accepted that promoter hypermethylation is associated with aberrant gene silencing in tumors [42,43]. To rule out epigenetic regulation of gene expression rather than regulation by transcription factors, we therefore disconnected all the edges from the genes in the tumor where there was an under-expression with a gain of promoter methylation.

As a result, we obtained 15 regulatory networks in which each gene member either had at least one incoming or outgoing edge. We found that the majority of the genes in the gene modules remained in the regulatory networks. Approximately 87% (13/15) of the networks contained more than 50% of the genes in the original modules, and 53.3% of the networks retained at least 80% of the genes in the modules. Eight of the regulatory networks consisted of more than 91% of the over-expressed genes, whereas five consisted of more than 91% of the under-expressed genes. The remaining two networks were mixed with up- and down-regulated genes and contained 41.85% and 25.22% of over-expressed genes, respectively. Pathway analysis showed that 14 out of the 15 networks were enriched by genes in at least one known cancer pathway as well as pathways that had not been recorded as cancer-related pathways in the literature, except network 10 (Table S1). Network 10 contained the highest percentage (28.9%) of lncRNAs compared to the other networks. For example, network 11 contained only one lncRNA (Figure S1), which may attribute to the absence of known cancer pathways in the network, as the function role of lncRNAs in cancer largely remains to be elucidated. We also conducted a bootstrapping experiment to examine the robustness of the regulatory networks in relation to noise. Out of 100 bootstrapping iterations, the median preserved rate of the edges in the 15 networks was 86.5% and 91.8% in at least 80 and 70 iterations, respectively. The majority of the edges in the networks demonstrated reasonable robustness to noise.

### 3.3. Key Regulatory Elements in the Networks

**Figure 2:**
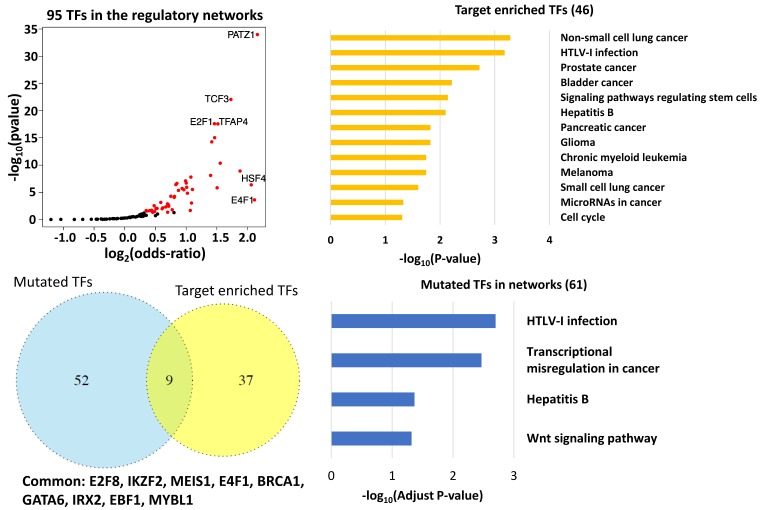
Key transcription factors in the network. (**a**) A total of 95 TFs demonstrated significant expression level alterations in lung adenocarcinomas and had at least one target gene in the same network. (**b**) The pathways that were significantly enriched for the 46 key TFs. (**c**) Nine common TFs were revealed by overlapping 46 key TFs with 61 TFs carrying at least one somatic mutation. (**d**) The pathways that were significantly enriched for 61 TFs harboring significant somatic mutation(s) in lung adenocarcinoma (LUAD). HTLV-1: human T cell lymphotropic virus type 1.

Of the 95 TFs that have at least one target gene in the same network, we found 46 (46/95) in which targeted genes were significantly enriched in the networks (*p* < 0.05 Fisher Exact Test, Figure 2a). We also calculated the odds ratios to measure how strong the presence of a specific TF’s target genes was in the network. *PATZ1*, *E4F1*, and *HSF4* had the largest odds ratios. Their odds ratios were larger than 4, suggesting that the presence of target genes inside the network are at least four times higher than those outside of it (Figure 2a). In a network, out-degree is defined as the number of outgoing edges emanating from a node. *PATZ1* (network 4, out-degree 192) is a DNA damage responsive TF that interacts with p53 [44]. The gene also regulates the expression of p53 target genes and is involved in cancer progression [45]. *E4F1* (network 8, out-degree 13) directly controls a transcriptional program involved in cell cycle checkpoints, metabolism, and mitochondrial homeostasis, as well as regulates the p53 response [46]. Inactivation of *HSF4* (network 8, out-degree 35), a heat shock factor, has been associated with tumorigenesis [44]. Additionally, the two TFs with the smallest *p*-values in the target gene enrichment test were also associated with tumors. *E2F1* (network 1, out-degree 147) can stimulate apoptosis and function as a tumor suppressor [47], while the *TCF3* fusion (network 4, out-degree 207) has been found in adenocarcinomas in situ [48].

The Database for Annotation, Visualization and Integrated Discovery (DAVID) pathway analysis suggested that the 46 TFs were significantly enriched in multiple cancer-related pathways (Benjamini *p*-adjust < 0.05, Figure 2b). These TFs were also prevalent in the measles and HTLV-I (human T cell lymphotropic virus type 1) infection pathways (*p*-adj = 2.6 × 10^−2^ and *p*-adj = 1.6 × 10^−2^, respectively). Measles virus has been used for cancer therapy [49], while HTLV-l plays a role in apoptosis [50]. Interestingly, the Hepatitis B pathway was also abundant with these TFs (*p*-adjust = 0.064, *p*-value = 7.9 × 10^−3^, respectively), suggesting a putative relationship between this disease and LUAD.

Sixty-one of 297 TFs in the network harbored at least one significant somatic mutation in LUAD. Nine of these mutated TFs, E2F8, IKZF2, MEIS1, E4F1, BRCA1, GATA6, IRX2, EBF1, and MYBL1, were also connected with a significant number of downstream targets in the network (Figure 2c, *p* < 0.05 Fisher Exact Test), suggesting their key regulatory roles in LUAD. The following literature review confirmed that six of the nine TFs, including E2F8, MEIS, E4F1, BRCA1, GATA6, and IRX2, play essential roles in lung cancer progression [46,51,52,53,54,55]. The genetic deletion of *EBF*1 is related to LUAD pathogenesis [56]. Its role in LUAD remains to be elucidated. *IKZF2* and *MYBL1* represent novel LUAD genes. Moreover, we found that the 61 significantly mutated TFs were enriched in four pathways, including transcriptional misregulation in cancer, Wnt signal pathway, Hepatitis B, and HTLV-1 infection pathway (Figure 2d). The latter two pathways were also enriched for the TFs revealed by gene set enrichment analysis (Figure 2b), highlighting the roles that the two pathways play in LUAD.

### 3.4. Key Regulatory Elements Outside the Networks

We further investigated TFs whose expression levels did not change significantly in LUAD; however, their downstream targets were significantly abundant in the disease networks (*p* < 0.05). We found that 63 TFs of this type also harbored one or more somatic mutation(s) (FDR < 0.05) in LUAD patients. Functional analysis suggested that the 63 TFs were abundant in cancer related pathways, including transcriptional misregulation in cancer, pathways in cancer, Hepatitis B, colorectal cancer, and pancreatic cancer (*p*-adjust < 0.04). The median number of networks potentially regulated by this type of TF was seven (Supplementary Figure S2).

**Figure 3:**
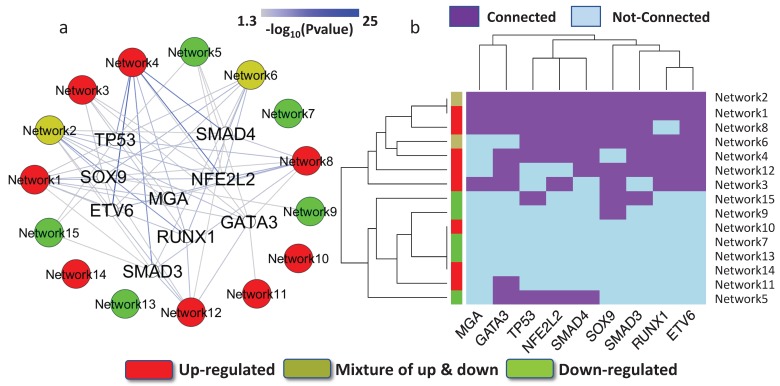
Key transcription factors outside the network. (**a**) The target genes of nine transcription factors that carried driver mutations were abundant in the regulatory networks. The color of the edge represents the *p*-value of the target gene enrichment analysis. The red-colored networks were over-expressed, whereas the green-colored networks were under-expressed in the disease. The yellow-colored networks were mixed with over-expressed and under-expressed genes. (**b**) The hierarchy clusters for the nine TFs and their regulated networks.

Previous studies have indicated that many identified mutations are related to cancer progression, but may only have an impact on tumor cells that have already emerged and on subsequent tumor growth [57]. This type of mutation is considered a passenger mutation. In contrast, mutations that cause cancer and promote tumor evolution are driver mutations. Hence, driver mutation information can further help us to prioritize key regulatory elements. OncoPrints is a function provided by cBioPortal, a widely used web tool in cancer research [58]. Coupling the driver mutations reported in OncoPrints with the results from target gene enrichment analysis, we identified nine critical transcription factors, encoded by *TP53*, *MGA*, *SOX9*, *ETV6*, *GATA3*, *NFE2L2*, *RUNX1*, *SMAD3*, and *SMAD4*. The nine genes that encode TFs appeared to be driver genes and acted as key network regulators. The collection of OncoPrints driver mutations consisted of a multiplicity of curated resources, including OncoKB, mutation hotspots, and recurrence in cBioPortal and COSMIC [59]. Each driver TF tends to mediate both under- and over-expressed networks (Figure 3a). We observed that the nine TFs clustered into several groups based on the networks that they potentially regulated (Figure 3b). For example, *TP53* and *NFE2L2* (also known as NRF2), were grouped together. It has been reported that p53 and NRF2 have similar functional roles, and both transcription factors enhance the capacity of cells to mitigate oxidative stress. It has also been found that NRF2 has an essential role in regulating p53 [60,61].

### 3.5. Regulation of Key lncRNAs

**Figure 4:**
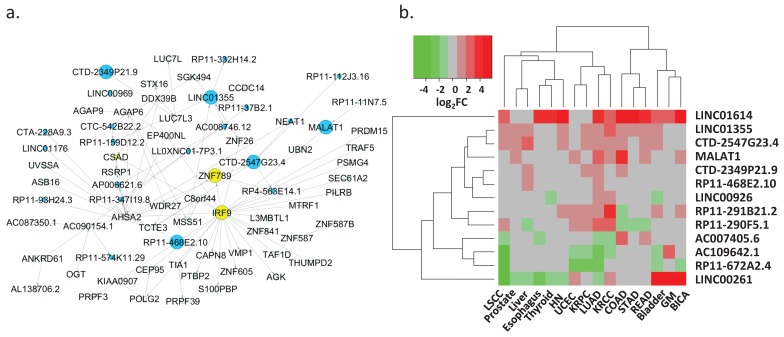
Key regulatory lncRNAs. (**a**) Network 10 consists of several lung cancer-related lncRNAs, such as MALAT1 and NEA1. The blue-colored nodes represent lncRNA transcripts. (**b**) The expression alterations (log_2_(FC)) of 13 key lncRNAs in the 16 solid tissue cancer types. Red denotes over-expression while green represents under-expression of the lncRNAs in cancers. Grey refers to an insignificant gene expression change.

We also identified several lncRNAs that may play a key regulatory role in LUAD. Of the 44 lncRNAs that were present in the networks (Figure 4a), 13 lncRNAs demonstrated great potential for binding protein-coding genes and controlling their transcription as inferred by their binding scores and Bayesian analysis predictions (Table 1). These key lncRNAs included several known lung cancer lncRNAs, such as metastasis-associated lung adenocarcinoma transcript (MALAT1), LINC00261, and LINC01614 (Table 1, top three rows) [62]. For example, MALAT1, a critical regulator of the metastasis phenotype in lung cancer cells, potentially regulated the expression of *UBN2* and NEAT1 (Figure 4b, Table 1, row two). NEAT1 is an oncogenic lncRNA, and its elevated expression level has been associated with the progression of non-small-cell lung cancer. In contrast, *UBN2* is a protein-coding gene that serves a transcriptional regulatory function.

We further examined the expression correlations of these lncRNAs and their corresponding target genes in 484 LUAD and 56 matched normal tissue samples, as well as in 53 human tissues based on Genotype-Tissue Expression (GTEx) project RNA-seq data (Table S2). The median value of Pearson correlation coefficients for the lncRNA and target gene pairs in normal lung and LUAD tissue samples was 0.77, which was consistent with the results from the co-expression module analysis, and 0.7 in the 53 human tissues. AC007405.6, LINC00261, and RP11-672A2.4, which are located within genomic proximity of their target genes, exhibited high expression correlation; the corresponding correlation coefficients were 0.83, 0.74, and 0.93 in normal lung and LUAD tissues and 0.81, 0.70 and 0.92 in the 53 human tissue samples, respectively. The results indicated putative *cis*-regulatory roles of these lncRNAs in controlling the expression of their target genes. Furthermore, these target genes are all known cancer-related genes [63,64]. For example, RP11-672A2.4 and its target gene *LRRC32* share the same promoter region. *LRRC32* encodes GARP, and aberrant expression of GARP has been reported in human breast, lung and colon cancers [64].

We expanded our investigation on these lncRNAs to 16 solid-tissue tumor types including LUAD in the TCGA project. Eight out of 13 key lncRNAs were differentially expressed in at least eight tumor types (|log2(FC)| > 1 & FDR < 0.05 Table 1, Figure 4b). LINC01614, CTD-2547G23.4, LINC01355, MALAT1, CTD-2349P21.9 and RP11-468E2.10 were over-expressed, whereas RP11-672A2.4 was under-expressed in cancers. The remaining six lncRNAs demonstrated mixed over-/under-expression patterns in different cancer types. Additionally, the lncRNA and target protein-coding gene correlation analysis suggested that some lncRNAs might promote tissue-specific regulation. For example, AC109642.1 and FMO2 were only correlatively expressed in LUAD and LSCC (Lung Squamous Cell Carcinoma). In contrast, the other lncRNAs showed correlated expression patterns with their targets in most cancer types (Supplementary Figure S3). Consistent with the expression analysis in the 53 human tissue samples, the expression of AC007405.6, LINC00261, and RP11-672A2.4 and their targets were correlated in these 16 cancer types.

Collectively, we confirmed three known cancer-associated lncRNAs and revealed nine other key lncRNAs that have yet to be reported in the literature and most likely have an essential regulatory role in LUAD (Table 1, Figure 4b).

## 4. Discussion

In this study, we integrated various types of genomic data to identify key regulatory elements in lung adenocarcinoma at the systems biology level. The genomic datasets, including whole-exome sequencing, RNA-seq, and DNA-methylation data, were obtained from the same patient cohort derived from the TCGA project. As cancer is a heterogeneous and complex disease, integrating genomics data from the same patient group can reduce the false positives that might arise from variations in individual genomic makeup rather than disease-related genetic alterations. We found that CHASM, a tool for disease driver predictions based on a random forest algorithm, yielded a large driver gene set with over 2000 genes being predicted as drivers at FDR < 0.05. On the other hand, VEST reported that the somatic mutations in over 12,000 genes had significant pathogenicity at FDR < 0.05. A majority of predicted driver genes (approximately 98%) by CHASM also showed significant pathogenicity by VEST. To pinpoint drivers with high specificity, we used a list of cancer driver genes that were experimentally verified and carefully curated to remove false positives. Our further regulation analysis of these genes in the network context offers novel insights into the mechanisms of the disease.

We revealed nine key TFs by combining co-expression modules, target gene enrichment, and somatic mutation analysis. The functional roles of six of these TFs in LUAD are supported by published studies. *E2F8* is a therapeutic target for lung cancer [48]. *MEIS1* has been found to inhibit non-small-cell lung cancer [52]. E4F1 has a critical role in cancer cell survival and could be a target for cancer therapy [46]. *BRCA1* is a breast-cancer-susceptibility gene, and a recent study indicated that this gene could be a potential molecular marker in non-small-cell lung cancer [53]. *GATA6* is an inhibitor of LUAD metastatic progression [54]. Hypermethylation of the *IRX2* promoter frequently occurs in LUAD [55]. *EBF*1, *IKZF2* and *MYBL1* are novel candidates that have regulatory roles in LUAD and could be used for further experimental validation. Our in-silico approach enables the integration of multi-dimensional experimental data to effectively infer key regulatory elements in the disease.

LncRNAs often have low expression levels, and the majority of lncRNAs lack sequence conservation [65,66]; consequently, most lncRNAs are not yet well characterized [10,67]. Although several lncRNAs have been studied in cancer research, much more work remains to be completed. Here, we used a very stringent threshold to define key lncRNAs in lung adenocarcinoma. Only 13 lncRNAs were reported as being key regulators of cancer by our study, and we might be underestimating the role of other lncRNAs. However, these lncRNAs could be well-defined targets for further experimental examination and help us gain new insights into lncRNA regulation in cancer.

Our study highlighted the association of Hepatitis B in lung adenocarcinoma development. Currently, only a few studies have focused on the hepatitis virus in cancer [68,69,70]. Our results along with other previous reports indicate that certain viral infections could serve as mechanisms for the initiation and progression of lung adenocarcinoma, necessitating further investigation regarding the contribution of viruses to lung carcinogenesis.

## 5. Conclusions

TFs and lnc RNAs are critical regulatory elements involved in lung cancer progression. The integrated analysis of multidimensional genomic data, including somatic mutations, gene expression, DNA methylation, TF-DNA interactions, and protein-lncRNA interactions, has enabled a deeper investigation into cancer development. Our study developed an integrative computational framework that applies network approaches to identify key regulatory elements that promote the initiation and progression of lung cancer. The regulatory networks were generated and refined by various genetic features. Key regulators revealed by multi-layer genomic data provided confident targets for other researchers for further experimental verification that could potentially be new targets for therapeutics and drug development.

## Figures and Tables

**Table 1:** Key regulatory long non-coding RNAs (lncRNAs), their corresponding downstream target protein-coding genes and lncRNAs, and the cancer types in which the key lncRNAs are differentially expressed.

Key lncRNA	Target Protein-Coding Gene(s) ^1^	Target lncRNA(s) ^1^	Differentially Expressed in Cancer Types ^2^
LINC00261	*FOXA2* (0.74; 0.7)	NA	BICA, Bladder, Esophagus, GM, HN, KRCC, Liver, LSCC, LUAD, Prostate, Thyroid, UCEC
MALAT1	*UBN2* (0.49; 0.83)	NEAT1 (0.77; 0.84)	BICA, COAD, KRCC, LUAD, Prostate, READ, UCEC
LINC01614	*TNFAIP6* (0.64; 0.64), *NOX4* (0.75; 0.71)	NA	BICA, Bladder, COAD, Esophagus, GM, HN, KRCC, LSCC, LUAD, READ, STAD, Thyroid
AC007405.6	*ERICH2* (0.83; 0.81)	NA	COAD, Esophagus, KRCC, LSCC, LUAD, READ
AC109642.1	*FMO2* (0.82; 0.81), *ANGPT1* (0.79; 0.77)	NA	Bladder, GM, KRCC, KRPC, LSCC, LUAD, UCEC
RP11-672A2.4	*LRRC32* (0.93; 0.92)	NA	BICA, Bladder, KRPC, LSCC, LUAD, UCEC
LINC01355	*WDR2* (0.72; 0.83), *SGK494* (0.67; 0.70), *CCDC14* (0.71; 0.84), *ZNF26* (0.66; 0.85), *PCGF3* (0.62; 0.72), *AC087350.1* (0.54; 0.72)	RP11-159D12.2 (0.68; 0.78), AC008746.12 (0.77; 0.85), RP11-332H14.2 (0.64; 0.65)	Bladder, COAD, HN, KRCC, KRPC, Liver, LSCC, LUAD, Prostate, READ, STAD
CTD-2547G23.4	*TCTE3* (0.78; 0.81), *HCG27* (0.71; 0.72)	NEAT1 (0.47; 0.70), LL0XNC01-7P3.1 (0.81; 0.82), LINC01355 (0.77; 0.85), RP4-563E14.1 (0.70; 0.82), RP11-112J3.16 (0.73; 0.72)	Bladder, COAD, HN, KRCC, KRPC, Liver, LSCC, LUAD, Prostate, READ
CTD-2349P21.9	*LUC7L3* (0.66; 0.65)	NA	COAD, KRPC, Liver, LUAD
RP11-468E2.10	*TCTE3* (0.65; 0.70)	NA	Liver, LUAD
LINC00926	*TRAF3IP3* (0.67; 0.70), *TNFRSF13C* (0.77; 0.80)*, FDCSP* (0.44; 0.38)	NA	Bladder, KRCC, LUAD, Thyroid
RP11-290F5.1	*FCRL5* (0.84; 0.84), *PIM2* (0.84; 0.77), *DERL3* (0.76; 0.66)	NA	COAD, KRCC, KRPC, Liver, LSCC, LUAD, READ, STAD, UCEC
RP11-291B21.2	*ZNF683* (0.80; 0.75)	AC002331.1 (0.74; 0.71)	BICA, Bladder, COAD, HN, KRCC, KRPC, LUAD, UCEC

^1^ The first number in parentheses represents the Pearson correlation coefficient of the key regulator and the corresponding target gene in the normal lung and lung adenocarcinoma (LUAD) tissues, while the second number represents their expression correlation in the 53 human tissues. ^2^ BICA: Breast Invasive Carcinoma; COAD: Colon Adenocarcinoma; HN: Head and Neck; GM: Glioblastoma Multiforme; KRCC: Kidney Renal Clear Cell Carcinoma; KRPC: Kidney Renal Papillary Cell Carcinoma; LSCC: Lung Squamous Cell Carcinoma; READ: Rectum Adenocarcinoma; STAD: Stomach Adenocarcinoma; UCEC: Uterine Corpus Endometrial Carcinoma.

## Supplementary Materials

The following are available online at www.mdpi.com/2073-4425/9/1/12/s1. Figure S1: The regulatory networks contained lncRNAs. (a) The percentage of lncRNAs in the networks. Network 10 appeared to contain the highest number of lncRNAs compared to the other networks. The lncRNAs were absent in five networks: 3, 4, 6, 12, and 15, respectively. (b) Network 11 contain only one lncRNA (blue) LINC01614, a known lung cancer lncRNA. The yellow box, RUNX2, is a key TF harboring lung cancer driver somatic mutation. TFs often regulate multiple genes in the network. For instance, the purple-colored nodes are potential target *PRRX1* genes. The purple circles represent the target PRRX1 genes reported by Marbach et al.; whereas, the purple diamonds represent the PRRX1 target genes identified by Bayesian network analysis. Figure S2: The number of significantly connected networks of mutated 63 TFs outside networks. Figure S3: The expression correlation of the key lncRNAs and the putative target genes in 16 solid-tissue cancer types. Table S1: The significantly enriched pathways of 15 gene regulatory network in lung adenocarcinomas. Table S2: The RNAseq of 53 human tissues used by GTEx project.
