# Erratum: Dan Li et al.; Transcription Factor and lncRNA Regulatory Networks Identify Key Elements in Lung Adenocarcinoma. *Genes* 2018, *9*, 12

**DOI:** 10.3390/genes9050251

**Published:** 2018-05-14

**Authors:** Dan Li, William Yang, Jialing Zhang, Jack Y. Yang, Renchu Guan, Mary Qu Yang

**Affiliations:** 1 Joint Bioinformatics Graduate Program, Department of Information Science, George W. Donaghey College of Engineering and Information Technology, University of Arkansas at Little Rock and University of Arkansas for Medical Sciences, 2801 S. University Ave, Little Rock, AR 72204, USA; dxli@ualr.edu (D.L.); wxyang1@ualr.edu (J.Y.Y.); rxguan@ualr.edu (R.G.); 2 School of Computer Science, Carnegie Mellon University, 5000 Forbes Ave, Pittsburgh, PA 15213, USA; wyang1@andrew.cmu.edu; 3 Department of Genetics, Yale University, New Haven, CT 06520, USA; jialing.zhang@yale.edu

The authors wish to make the following change to their paper [1]. In the Acknowledgments Section, “This study was supported in part by United States National Institutes of Health (NIH) Academic Research Enhancement Award 1R15GM114739 and National Institute of General Medical Sciences (NIH/NIGMS) 5P20GM103429, United States Food and Drug Administration (FDA) HHSF223201510172C through Arkansas Research Alliance (ARA) BAA-15-00121 and Arkansas Science and Technology Authority (ASTA) Basic Science Research 15-B-23 and 15-B-38” should read as “This study was partially supported by United States National Institutes of Health (NIH) Academic Research Enhancement Award 1R15GM114739 and National Institute of General Medical Sciences (NIH/NIGMS) 5P20GM103429, Arkansas Science and Technology Authority (ASTA) Basic Science Research 15-B-23 and 15-B-38 and the Food and Drug Administration (FDA), contract No. HHSF223201510172C and HHSF223201610111C. However, the information contained herein represents the position of the author(s) and not necessarily that of the NIH and FDA.”

The authors would like to apologize for any inconvenience caused. The change does not affect the scientific results. The manuscript will be updated, and the original will remain online on the article webpage.
