# Supplementary material for: Transcription Factor and lncRNA Regulatory Networks Identify Key Elements in Lung Adenocarcinoma
Source: Genes (Basel). 2018 Jan 5;9(1):12. doi: 10.3390/genes9010012 (PMC5793165; doi:10.3390/genes9010012)
Supplement: Supplementary file 1 [file genes-09-00012-s001.docx]

Article

Transcription Factor and lncRNA Regulatory Networks Identify Key Elements in Lung Adenocarcinoma

Dan Li ^1^, William Yang ^2^, Jialing Zhang ^3^, Renchu Guan ^1^ and Mary Qu Yang ^1,^*

Supplementary Materials

**Figure S1.** The regulatory networks contained lncRNAs. (**a**) The percentage of lncRNAs in the networks. Network 10 appeared to contain the highest number of lncRNAs compared to the other networks. The lncRNAs were absent in five networks: 3, 4, 6, 12, and 15, respectively. (**b**) Network 11 contain only one lncRNA (blue) *LINC01614*, a known lung cancer lncRNA. The yellow box, *RUNX2*, is a driver TF harboring lung cancer driver somatic mutations. TFs often regulate multiple genes in the network. For instance, the purple-colored nodes are potential target *PRRX1* genes. The purple circles represent the target PRRX1genes reported by Marbach et al.; whereas, the purple diamonds represent the PRRX1 target genes identified by Bayesian network analysis.

**Figure S2.** The number of significantly connected networks of mutated 63 TFs outside networks.

**Figure S3.** The expression correlation of the key lncRNAs and the putative target genes in 16 solid-tissue cancer types.

**Table S1.** The significantly enriched pathways of 15 gene regulatory network in lung adenocarcinomas. The Column VI in the table shows the published papers that demonstrate the involvement of the signaling pathway (Column I) in cancer.

| KEGG Pathways | *p*-value | Benjamini | References |
| --- | --- | --- | --- |
| Network 1 | | | |
| Cell cycle | 5.20E-35 | 6.80E-33 | Role of cell cycle regulators in lung carcinogenesis. *Cell adhesion & migration, 2010* |
| DNA replication | 1.50E-22 | 1.00E-20 | Non-small cell lung cancer exhibits transcript overexpression of genes associated with homologous recombination and DNA replication pathways. *Cancer research, 2009* |
| Fanconi anemia pathway | 2.20E-08 | 9.80E-07 |  |
| Oocyte meiosis | 1.60E-07 | 5.20E-06 |  |
| Mismatch repair | 5.40E-07 | 1.40E-05 |  |
| p53 signaling pathway | 2.00E-05 | 4.30E-04 |  |
| Progesterone-mediated oocyte maturation | 2.70E-05 | 5.10E-04 |  |
| Base excision repair | 8.90E-05 | 1.50E-03 | Polymorphisms in DNA Base Excision Repair Genes ADPRT and XRCC1 and Risk of Lung Cancer. *Cancer Research, 2005* |
| Pyrimidine metabolism | 1.30E-04 | 1.90E-03 |  |
| HTLV-I infection | 2.50E-04 | 3.20E-03 |  |
| Nucleotide excision repair | 6.60E-04 | 7.90E-03 |  |
| One carbon pool by folate | 9.80E-04 | 1.10E-02 |  |
| RNA transport | 2.00E-03 | 2.00E-02 |  |
| Purine metabolism | 2.40E-03 | 2.20E-02 |  |
| Small cell lung cancer | 3.30E-03 | 2.80E-02 |  |
| Homologous recombination | 4.10E-03 | 3.30E-02 |  |
| Network 2 | | | |
| Glycolysis/Gluconeogenesis | 2.40E-04 | 4.00E-02 |  |
| Biosynthesis of antibiotics | 6.10E-04 | 5.10E-02 |  |
| Biosynthesis of amino acids | 2.60E-03 | 1.40E-01 |  |
| Carbon metabolism | 5.30E-03 | 2.00E-01 | One-carbon metabolism in cancer. *British journal of cancer, 2017* |
| ECM-receptor interaction | 5.70E-03 | 1.80E-01 |  |
| PI3K-Akt signaling pathway | 2.90E-02 | 5.60E-01 | Tyrosine kinase—Role and significance in Cancer. *International Journal of Medical Sciences, 2004* |
| Axon guidance | 3.20E-02 | 5.50E-01 |  |
| HIF-1 signaling pathway | 3.70E-02 | 5.50E-01 |  |
| Fc gamma R-mediated phagocytosis | 7.30E-02 | 7.60E-01 |  |
| Vibrio cholerae infection | 7.70E-02 | 7.50E-01 |  |
| Network 3 | | | |
| Ribosome biogenesis in eukaryotes | 4.20E-03 | 3.70E-01 |  |
| Protein processing in endoplasmic reticulum | 1.70E-02 | 6.20E-01 |  |
| Fanconi anemia pathway | 2.70E-02 | 6.30E-01 | Exploiting the Fanconi Anemia Pathway for Targeted Anti-Cancer Therapy. *Molecules and cells, 2015* |
| Network 4 | | | |
| Glycosaminoglycan biosynthesis-chondroitin sulfate/dermatan sulfate | 2.00E-03 | 2.50E-01 |  |
| RIG-I-like receptor signaling pathway | 1.20E-02 | 5.90E-01 | Cancer therapies activate RIG-I-like receptor pathway through endogenous non-coding RNAs. *Oncotarget, 2016* |
| Glycosaminoglycan biosynthesis-heparan sulfate/heparin | 3.80E-02 | 8.40E-01 |  |
| Network 5 | | | |
| Malaria | 1.50E-03 | 2.20E-01 |  |
| Cell adhesion molecules (CAMs) | 3.20E-03 | 2.30E-01 | Cancer Cell Adhesion and Metastasis: Selectins, Integrins, and the Inhibitory Potential of Heparins. *International Journal of Cell Biology, 2012* |
| Leukocyte transendothelial migration | 1.60E-02 | 6.00E-01 |  |
| Rap1 signaling pathway | 2.90E-02 | 7.00E-01 |  |
| Network 6 | | | |
| Alzheimer’s disease | 1.90E-05 | 3.20E-03 |  |
| Huntington’s disease | 3.10E-04 | 2.60E-02 |  |
| cGMP-PKG signaling pathway | 4.60E-04 | 2.60E-02 |  |
| Oxidative phosphorylation | 4.90E-04 | 2.10E-02 | Waves of gene regulation suppress and then restore oxidative phosphorylation in cancer cells. *The international journal of biochemistry & cell biology, 2011* |
| Parkinson’s disease | 3.50E-03 | 1.10E-01 |  |
| Non-alcoholic fatty liver disease (NAFLD) | 4.90E-03 | 1.30E-01 |  |
| Focal adhesion | 7.60E-03 | 1.70E-01 |  |
| Purine metabolism | 1.10E-02 | 2.10E-01 |  |
| Melanoma | 1.70E-02 | 2.80E-01 |  |
| Rap1 signaling pathway | 2.60E-02 | 3.70E-01 | Roles of Rap1 signaling in tumor cell migration and invasion. *Cancer biology & medicine, 2017* |
| Metabolic pathways | 2.70E-02 | 3.50E-01 |  |
| Platelet activation | 3.40E-02 | 3.90E-01 |  |
| Endometrial cancer | 3.50E-02 | 3.80E-01 |  |
| Signaling pathways regulating pluripotency of stem cells | 4.50E-02 | 4.30E-01 |  |
| Glucagon signaling pathway | 4.80E-02 | 4.40E-01 |  |
| Network 7 | | | |
| Staphylococcus aureus infection | 1.20E-26 | 2.00E-24 | Risk factors for mortality caused by Staphylococcus aureus bacteremia in cancer patients. *Enfermedades infecciosas y microbiología clínica, 2010* |
| Leishmaniasis | 6.90E-22 | 5.60E-20 |  |
| Phagosome | 1.50E-18 | 8.30E-17 |  |
| Tuberculosis | 6.50E-17 | 4.60E-15 |  |
| Osteoclast differentiation | 1.50E-14 | 5.00E-13 | Cancer-associated osteoclast differentiation takes a good look in the miR(NA)ror. *Cancer Cell, 2014* |
| Graft-versus-host disease | 2.50E-13 | 6.70E-12 |  |
| Rheumatoid arthritis | 3.50E-12 | 8.20E-11 |  |
| Type I diabetes mellitus | 6.70E-12 | 1.40E-10 |  |
| Allograft rejection | 3.20E-11 | 5.90E-10 |  |
| Antigen processing and presentation | 7.10E-11 | 1.20E-09 |  |
| Asthma | 7.30E-11 | 1.10E-09 |  |
| Viral myocarditis | 3.40E-10 | 4.60E-09 |  |
| Intestinal immune network for IgA production | 5.70E-10 | 7.10E-09 |  |
| Cell adhesion molecules (CAMs) | 7.00E-10 | 8.20E-09 |  |
| Inflammatory bowel disease (IBD) | 1.40E-09 | 1.50E-08 |  |
| Autoimmune thyroid disease | 1.80E-09 | 1.80E-08 |  |
| Systemic lupus erythematosus | 2.40E-09 | 2.30E-08 | Cancer complicating systemic lupus erythematosus – a dichotomy emerging from a nested case-control study. *Lupus, 2013* |
| Toxoplasmosis | 2.80E-08 | 2.50E-07 |  |
| Herpes simplex infection | 2.10E-07 | 1.80E-06 |  |
| Influenza A | 3.50E-06 | 2.80E-05 |  |
| Network 8 | | | |
| Glycerophospholipid metabolism | 2.40E-03 | 8.40E-02 | Lipid Metabolism, Apoptosis and Cancer Therapy. *International journal of molecular sciences, 2015* |
| Network 9 | | | |
| Natural killer cell mediated cytotoxicity | 2.30E-07 | 3.80E-05 | Sensitization of human breast cancer cells to natural killer cell-mediated cytotoxicity by proteasome inhibition. *Clinical and experimental immunology, 2009* |
| T cell receptor signaling pathway | 4.30E-07 | 3.50E-05 |  |
| Platelet activation | 3.70E-06 | 2.00E-04 |  |
| Fc epsilon RI signaling pathway | 1.80E-05 | 7.10E-04 |  |
| B cell receptor signaling pathway | 1.90E-05 | 6.20E-04 | The B-cell receptor signaling pathway as a therapeutic target in CLL. *Blood, 2012* |
| Osteoclast differentiation | 2.90E-05 | 7.90E-04 |  |
| Measles | 3.30E-05 | 7.60E-04 |  |
| VEGF signaling pathway | 9.40E-05 | 1.90E-03 | The VEGF signaling pathway in cancer: the road ahead. *Chinese journal of cancer, 2013* |
| Inflammatory mediator regulation of TRP channels | 1.90E-04 | 3.30E-03 |  |
| Jak-STAT signaling pathway | 3.80E-04 | 6.10E-03 |  |
| Ras signaling pathway | 4.20E-04 | 6.10E-03 |  |
| Leukocyte transendothelial migration | 5.80E-04 | 7.80E-03 |  |
| NF-kappa B signaling pathway | 6.60E-04 | 8.20E-03 | The complexity of NF-κB signaling in inflammation and cancer. *Molecular cancer, 2013* |
| Primary immunodeficiency | 7.50E-04 | 8.60E-03 |  |
| Rap1 signaling pathway | 1.00E-03 | 1.10E-02 |  |
| Cell adhesion molecules (CAMs) | 1.70E-03 | 1.70E-02 |  |
| Chemokine signaling pathway | 1.90E-03 | 1.80E-02 |  |
| Hepatitis B | 2.00E-03 | 1.70E-02 |  |
| Network 10 | | | |
| NA |  |  |  |
| Network 11 | | | |
| ECM-receptor interaction | 9.70E-13 | 5.20E-11 | The extracellular matrix: A dynamic niche in cancer progression. *The Journal of Cell Biology, 2012* |
| Protein digestion and absorption | 4.70E-11 | 1.30E-09 |  |
| Focal adhesion | 5.40E-09 | 9.80E-08 |  |
| PI3K-Akt signaling pathway | 7.20E-07 | 9.80E-06 |  |
| Amoebiasis | 4.00E-06 | 4.40E-05 |  |
| Platelet activation | 1.30E-05 | 1.20E-04 |  |
| Network 12 | | | |
| Spliceosome | 2.50E-02 | 2.80E-01 | The spliceosome is a therapeutic vulnerability in MYC-driven cancer. *Nature, 2015* |
| Ribosome | 2.60E-02 | 1.60E-01 |  |
| Network 13 | | | |
| Purine metabolism | 3.70E-03 | 2.20E-02 | A New View into the Regulation of Purine Metabolism: The Purinosome. *Trends in Biochemical Sciences, 2017* |
| Network 14 | | | |
| Cytokine-cytokine receptor interaction | 1.70E-10 | 1.20E-08 |  |
| Chemokine signaling pathway | 6.50E-07 | 2.30E-05 |  |
| Primary immunodeficiency | 8.80E-04 | 2.10E-02 |  |
| NF-kappa B signaling pathway | 1.40E-03 | 2.50E-02 | The complexity of NF-κB signaling in inflammation and cancer. *Molecular cancer, 2013* |
| Toll-like receptor signaling pathway | 2.80E-03 | 4.00E-02 |  |
| Network 15 | | | |
| Vascular smooth muscle contraction | 3.90E-03 | 1.90E-01 |  |
| Proteoglycans in cancer | 1.60E-02 | 3.60E-01 | Proteoglycans in cancer biology, tumour microenvironment and angiogenesis. *Journal of cellular and molecular medicine, 2011* |
| Leukocyte transendothelial migration | 4.10E-02 | 5.30E-01 |  |

**Table S2.** The RNAseq of 53 tissues used by GTEx project.

| Adipose—Subcutaneous | Brain—Spinal cord (cervical c-1) | Minor Salivary Gland |
| --- | --- | --- |
| Adipose—Visceral (Omentum) | Brain—Substantia nigra | Muscle—Skeletal |
| Adrenal Gland | Breast—Mammary Tissue | Nerve—Tibial |
| Artery—Aorta | Cells—EBV-transformed lymphocytes | Ovary |
| Artery—Coronary | Cells—Transformed fibroblasts | Pancreas |
| Artery—Tibial | Cervix—Ectocervix | Pituitary |
| Bladder | Cervix—Endocervix | Prostate |
| Brain—Amygdala | Colon—Sigmoid | Skin—Not Sun Exposed (Suprapubic) |
| Brain—Anterior cingulate cortex (BA24) | Colon—Transverse | Skin—Sun Exposed (Lower leg) |
| Brain—Caudate (basal ganglia) | Esophagus—Gastroesophageal Junction | Small Intestine—Terminal Ileum |
| Brain—Cerebellar Hemisphere | Esophagus—Mucosa | Spleen |
| Brain—Cerebellum | Esophagus—Muscularis | Stomach |
| Brain—Cortex | Fallopian Tube | Testis |
| Brain—Frontal Cortex (BA9) | Heart—Atrial Appendage | Thyroid |
| Brain—Hippocampus | Heart—Left Ventricle | Uterus |
| Brain—Hypothalamus | Kidney—Cortex | Vagina |
| Brain—Nucleus accumbens (basal ganglia) | Liver | Whole Blood |
| Brain—Putamen (basal ganglia) | Lung |  |
